# Capnodynamic monitoring of lung volume and blood flow in response to increased positive end-expiratory pressure in moderate to severe COVID-19 pneumonia: an observational study

**DOI:** 10.1186/s13054-022-04110-0

**Published:** 2022-07-31

**Authors:** Luis Schulz, Antony Stewart, William O’Regan, Peter McCanny, Danielle Austin, Magnus Hallback, Mats Wallin, Anders Aneman

**Affiliations:** 1 Intensive Care Unit, Liverpool Hospital, South Western Sydney Local Health District, Locked Bag 7103, Liverpool BC, NSW 1871 Australia; 2 Maquet Critical Care AB, Solna, Sweden; 3 Department of Physiology and Pharmacology, Karolinska Institute, Stockholm, Sweden; 4 SouthWestern Clinical School, University of New South Wales, Sydney, Australia; 5 Ingham Institute for Applied Medical Research, Liverpool, NSW Australia

**Keywords:** COVID-19, Mechanical ventilation, Positive end-expiratory pressure, Lung volume, Lung perfusion, Monitoring

## Abstract

**Background:**

The optimal level of positive end-expiratory pressure (PEEP) during mechanical ventilation for COVID-19 pneumonia remains debated and should ideally be guided by responses in both lung volume and perfusion. Capnodynamic monitoring allows both end-expiratory lung volume (${\text{EELV}}_{{{\text{CO}}_{2} }}$) and effective pulmonary blood flow (EPBF) to be determined at the bedside with ongoing ventilation.

**Methods:**

Patients with COVID-19-related moderate to severe respiratory failure underwent capnodynamic monitoring of ${\text{EELV}}_{{{\text{CO}}_{2} }}$ and EPBF during a step increase in PEEP by 50% above the baseline (PEEP_low_ to PEEP_high_). The primary outcome was a > 20 mm Hg increase in arterial oxygen tension to inspired fraction of oxygen (*P*/*F*) ratio to define responders versus non-responders. Secondary outcomes included changes in physiological dead space and correlations with independently determined recruited lung volume and the recruitment-to-inflation ratio at an instantaneous, single breath decrease in PEEP. Mixed factor ANOVA for group mean differences and correlations by Pearson’s correlation coefficient are reported including their 95% confidence intervals.

**Results:**

Of 27 patients studied, 15 responders increased the *P*/*F* ratio by 55 [24–86] mm Hg compared to 12 non-responders (*p* < 0.01) as PEEP_low_ (11 ± 2.7 cm H_2_O) was increased to PEEP_high_ (18 ± 3.0 cm H_2_O). The ${\text{EELV}}_{{{\text{CO}}_{2} }}$ was 461 [82–839] ml less in responders at PEEP_low_ (*p* = 0.02) but not statistically different between groups at PEEP_high_. Responders increased both ${\text{EELV}}_{{{\text{CO}}_{2} }}$ and EPBF at PEEP_high_ (*r* = 0.56 [0.18–0.83], *p* = 0.03). In contrast, non-responders demonstrated a negative correlation (*r* = − 0.65 [− 0.12 to − 0.89], *p* = 0.02) with increased lung volume associated with decreased pulmonary perfusion. Decreased (− 0.06 [− 0.02 to − 0.09] %, *p* < 0.01) dead space was observed in responders. The change in ${\text{EELV}}_{{{\text{CO}}_{2} }}$ correlated with both the recruited lung volume (*r* = 0.85 [0.69–0.93], *p* < 0.01) and the recruitment-to-inflation ratio (*r* = 0.87 [0.74–0.94], *p* < 0.01).

**Conclusions:**

In mechanically ventilated patients with moderate to severe COVID-19 respiratory failure, improved oxygenation in response to increased PEEP was associated with increased end-expiratory lung volume and pulmonary perfusion. The change in end-expiratory lung volume was positively correlated with the lung volume recruited and the recruitment-to-inflation ratio. This study demonstrates the feasibility of capnodynamic monitoring to assess physiological responses to PEEP at the bedside to facilitate an individualised setting of PEEP.

*Trial registration*: NCT05082168 (18th October 2021).

**Supplementary Information:**

The online version contains supplementary material available at 10.1186/s13054-022-04110-0.

## Background

The selection of level of positive end-expiratory pressure (PEEP) during invasive mechanical ventilatory support for COVID-19 pneumonia remains debated. A low level PEEP (≤ 10 cm H_2_O) for COVID-19-related acute respiratory failure was supported by a third of experts in a recent Delphi method consensus statement with half of the panel members remaining neutral without agreement on PEEP titration [1]. In contrast, the updated Surviving Sepsis Campaign guidelines for COVID-19 provided a strong recommendation to use high level of PEEP (> 10 cm H_2_O) in moderate to severe acute respiratory distress syndrome associated with COVID-19 (C-ARDS) [2]. The static compliance of the respiratory system was highly variable in a large international cohort study of mechanically ventilated COVID-19 patients [3]. This highlights the importance of adequate monitoring to allow for an individualised PEEP strategy as an identical level of PEEP might either lead to lung recruitment or overdistention depending on the compliance state. The gas exchange abnormalities in C-ARDS result from a range of ventilation/perfusion inequalities with further complexity added by diverse changes over time and in different lung regions [4, 5]. The responses to different levels of PEEP can be expected to be equally diverse and should ideally be guided by assessment of lung recruitability and lung perfusion. The effects of incremental PEEP on aerated lung tissue in C-ARDS have been investigated using computed tomography [6, 7], electrical impedance tomography [8, 9] and lung ultrasound [10]. These techniques require both specialised equipment and procedural expertise. In contrast, capnodynamic monitoring of lung volume and perfusion may be integrated with standard ventilators at the bedside and provides continuous measurements without special respiratory manoeuvres or interruptions [11–14]. This study aimed to assess the feasibility of capnodynamic monitoring of the responses to increased PEEP on gas exchange in mechanically ventilated patients with moderate to severe C-ARDS. It was hypothesised that in patients responding with increased arterial oxygen tension to inspired fraction of oxygen ratio at high PEEP, capnodynamic monitoring would identify an increase in lung volume with increased or preserved pulmonary blood flow.

## Methods

This pragmatic, prospective, observational open study was approved by the South Western Sydney Local Health District Human Research Ethics Committee (2020/ETH00778) and registered on ClinicalTrials.gov (NCT05082168). Patients admitted to Liverpool Hospital ICU between September 2021 and February 2022 were screened for eligibility with verbal consent from the patient’s person responsible. The study is reported as per the STROBE guidelines for observational studies [15] (Additional File 1: Table S1).

### Patient management and eligibility

All patients were positive for SARS-Cov2 RNA in real-time PCR assay of a nasopharyngeal swab. Patients received continuous sedation and analgesia and in case of persistent patient-ventilator dyssynchrony, neuromuscular blockade was established. Lung protective ventilation at a tidal volume (*V*_t_) of 6 ml/kg predicted body weight (PBW) with plateau pressures (*P*_plat_) < 30 cm H_2_O and a respiratory rate (RR) adjusted for permissive hypercarbia (pH > 7.25) was used, with the inspired fraction of oxygen (F_i_O_2_) titrated to a peripheral oxygen saturation (S_p_O_2_) of 88–92%. A pressure-regulated volume-controlled mode was used (Draeger V500, Draeger, Lubeck, Germany or Hamilton C6, Hamilton Medical AG, Bonaduz, Switzerland). The study inclusion criteria were (1) patient identified within 72 h of admission to ICU for confirmed SARS-CoV-2 pneumonia; (2) age > 18 years; (3) moderate or severe ARDS (a ratio of partial pressure of oxygen in arterial blood (P_a_O_2_) to inspired fraction of oxygen (F_i_O_2_) ≤ 200 mm Hg) and receiving invasive ventilatory support with ≥ 5 cm H_2_O PEEP; (4) fully synchronised with the ventilator; and (5) a recruitment manoeuvre by increasing PEEP to + 50% above the set level warranted in the opinion of the treating clinical team, independent of the study protocol. Exclusion criteria were (1) pneumoperitoneum; (2) pneumomediastinum; (3) undrained pneumothorax or ongoing air leak; (4) haemodynamic instability (> 30% increase in vasopressor over last 6 h or noradrenaline > 0.5 µg/kg/min).

### Study procedures

In eligible patients, the endotracheal tube was temporarily clamped in end-inspiration and the standard ventilator changed to the research Servo-I ventilator (Maquet Critical Care, Solna, Sweden) with the F_i_O_2_, *V*_t_ and PEEP settings unchanged. Patients were ventilated in a volume-controlled mode with a modified breathing pattern in which short expiratory holds were added to 3 out of 9 consecutive breaths to cause cyclical changes in the alveolar partial pressure of CO_2_ of at least 3 mm Hg. The RR was adjusted to maintain an overall effective RR with the minute ventilation unchanged. The capnodynamic algorithm to derive end-expiratory lung volume (${\text{EELV}}_{{{\text{CO}}_{2} }}$) and effective, i.e. non-shunted, pulmonary blood flow (EPBF) has been described in detail elsewhere [11, 14, 16]. In brief, volumetric capnograms are created in real time combining data from a mainstream infrared CO_2_ sensor (Capnostat®, Philips Respironics, Philadelphia, PA, USA) and the integrated flow signal in the Servo-I ventilator. Data were exported to a laptop running dedicated software (MATLAB®, Mathworks, Natick, MA, USA) to obtain real-time measurements of ${\text{EELV}}_{{{\text{CO}}_{2} }}$ and EPBF (see below, *Calculations*). The validity of EPBF [13, 16] and ${\text{EELV}}_{{{\text{CO}}_{2} }}$ [11, 14] against standard methods to monitor cardiac output and functional residual capacity have been reported in clinical studies. After 20 min of baseline PEEP (PEEP_low_) recording, an arterial blood gas was obtained and analysed immediately (GEM Premier 5000, Artarmon, New South Wales, Australia). The PEEP was then increased by 50% above baseline level (PEEP_high_) and a repeat arterial blood gas analysis performed after 20 min. The PEEP_high_ was then instantaneously reduced to PEEP_low_ within one breath during a prolonged (5 s) expiration to assess the exhaled tidal volume. The recruited lung volume and the recruitment-to-inflation ratio (*R*/*I* ratio) were derived as described below, *Calculations*. After completion of study procedures, the endotracheal tube was temporarily clamped and the patient was reconnected to the standard ventilator. Study procedures and data were open to the clinical team, and at their discretion, an increased PEEP above the pre-study level was considered in responders.

Patient and clinical characteristics were recorded prior to changing the ventilator. Ventilator data including the ${\text{EELV}}_{{{\text{CO}}_{2} }}$ and EPBF were subsequently analysed offline using custom software (www.icumaps.org/visualizer) that calculated the mean values over 30 breaths. The data are reported for the end of baseline PEEP_low_ and the end of PEEP_high_.

### Calculations

The predicted body weight was calculated according to [17]. The physiological dead space (*V*_d_/*V*_t_) was calculated according to the Enghoff equation [18] based on arterial partial pressure of CO_2_ (P_a_CO_2_) and end-tidal CO_2_ (ET-CO_2_). The static compliance of the respiratory system (*C*_rs_) was calculated as the *V*_t_ divided by the *P*_plat_ − PEEP difference, with the latter difference representing the driving pressure, *P*_dr_. The recruited lung volume by the PEEP manoeuvre was assessed as described by Chen et al. [19] during a single breath exhalation. The PEEP_high_ was instantaneously decreased to the patient’s PEEP_low_ in a prolonged expiration and the actual change of end-expiratory lung volume was determined from the difference in expiratory tidal volume before and during the PEEP reduction.

The expected change of end-expiratory lung volume was calculated as the product of the *C*_rs_ at PEEP_low_ and the difference in PEEP (PEEP_high_ − PEEP_low_). The recruited lung volume (∆Vol_rec_) was calculated as the difference between the actual and the expected end-expiratory lung volumes at the rapid PEEP reduction. The recruitment-to-inflation ratio (*R*/*I* ratio) was calculated as previously described [19] dividing the compliance of the recruited lung volume by the *C*_rs_ at PEEP_low_.

The capnodynamic method is based on the differential Fick equation for carbon dioxide [12]. With the assumption that the lung volume, pulmonary blood flow and the mixed venous content of CO_2_ (C_v_CO_2_) remain constant during each 9-breath measurement cycle, the created variability in expired CO_2_ makes it possible to solve the nine capnodynamic equations with the least square method to obtain the three unknown parameters: ${\text{EELV}}_{{{\text{CO}}_{2} }}$, C_v_CO_2_ and EPBF. The capnodynamic equation describes a mole balance of CO_2_ between the transport of CO_2_ to and from the lungs and the rate of change in the CO_2_ content of the lungs and is expressed as

$${\text{EELV}}_{{{\text{CO}}_{2} }} \cdot \left( {{\text{F}}_{{\text{A}}} {\text{CO}}_{2}^{n} - {\text{F}}_{{\text{A}}} {\text{CO}}_{2}^{n - 1} } \right) = {\text{EPBF}} \cdot \Delta t^{n} \cdot \left( {{\text{C}}_{{\text{v}}} {\text{CO}}_{2} - {\text{C}}_{{\text{c}}} {\text{CO}}_{2}^{n} } \right) - {\text{V}}_{{\text{t}}} {\text{CO}}_{2}^{n}$$

The F_A_CO_2_ represents the mean alveolar fraction of CO_2_ measured at the mid-point of the slope of phase III of the volumetric capnogram [20], *n* is the current breath, *n* − 1 is the previous breath, ∆*t*^*n*^ is the current breath cycle time, C_c_CO_2_^*n*^ is the content of CO_2_ in the pulmonary capillary blood calculated from F_A_CO_2_ and *V*_t_CO_2_^*n*^ is the volume of CO_2_ eliminated by a breath. The capnodynamic equation system is applied in a continuous breath-by-breath fashion where every 10th breath is replacing the first one in the nine-breath cycle.

### Outcomes

Changes in ${\text{EELV}}_{{{\text{CO}}_{2} }}$ and EPBF were assessed against the primary outcome of a change in P_a_O_2_/F_i_O_2_ ratio induced by increased PEEP. An increase in P_a_O_2_/F_i_O_2_ > 20 mm Hg was used to classify responders versus non-responders [21]. The secondary outcomes included *V*_d_/*V*_t_ and correlations to changes in ${\text{EELV}}_{{{\text{CO}}_{2} }}$ and EBPF, and ∆Vol_recr_ as well as *R*/*I* ratio and correlations to changes in ${\text{EELV}}_{{{\text{CO}}_{2} }}$.

### Statistics

No formal sample size calculation was performed for this observational study, and the final sample size was determined by the number of patients admitted to ICU during two surges of the COVID-19 pandemic in south-western Sydney, Australia. Continuous data are presented as mean ± standard deviation (SD) or median [interquartile range, IQR] for normally and non-normally distributed data as determined by the D’Agostino–Pearson normality test. A mixed factor ANOVA with PEEP set as the within-subjects effect and the *P*/*F* ratio response set as the between-subjects effect was performed with Greenhouse–Geisser correction for homogeneity of variance. Post hoc testing was performed with Bonferroni correction for repeated measurements and the main effects reported as median differences with their 95% confidence interval for significant findings. Correlations are reported with Pearson’s *r* and regressions shown including the 95% confidence intervals from 1000 bootstraps. Statistical significance was set at a two-sided *p* value < 0.05. Data were analysed using the R statistical software (version 4.0.3, R Foundation for Statistical Computing, Vienna, Austria) with graphs generated using GraphPad PRISM (version 9.3.1, San Diego, CA, USA).

## Results

A total of 94 patients with moderate or severe C-ARDS were invasively ventilated in ICU during the study time period with 29 patients enrolled in the study. The study protocol was abandoned in one patient at a P_a_O_2_/F_i_O_2_ ratio of 69 with decision to proceed to veno-venous ECMO. Severe hypercarbia precluded achieving an appropriate cyclic ET-CO_2_ change for capnodynamic measurements in one patient. Hence, 27 patients were investigated and included in this report. The P_a_O_2_/F_i_O_2_ ratio increased > 20 mm Hg from PEEP_low_ to PEEP_high_ in 15 patients (responders), while such an improvement was absent in 12 patients (non-responders). The patient characteristics are reported in Table 1. No significant differences between P_a_O_2_/F_i_O_2_ responders and non-responders were noted at PEEP_low_ for gas exchange and pulmonary mechanics (Table 2). The PEEP_high_ manoeuvre increased P_a_O_2_ in responders (mean difference 33 [22–44] mm Hg, *p* < 0.001) with an increase in P_a_O_2_/F_i_O_2_ ratio (mean difference 57 [36–78] mm Hg, *p* = 0.001) (Table 2). The *C*_rs_ was greater (mean difference 8.5 [3.2–16] mL/cm H_2_O, *p* = 0.01) in responders at PEEP_high_ with a corresponding decrease in *P*_dr_ (mean difference 6.5 [5.5–11] cm H_2_O, *p* = 0.005) (Table 2).

**Table 1:** General characteristics of the study cohort

Variables	Population (*n* = 27)
Male (*n*, %)	20 (74%)
Age (years)	52.5 ± 13
Height (cm)	171 ± 9.5
BMI (kg/m^2^)	35.8 ± 8.4
Diabetes (*n*, %)	6 (22%)
Hypertension (*n*, %)	6 (22%)
APACHE III	57 ± 19
Time since diagnosis (days)	6 [3–8]
Time since hospital admission (days)	6 [2–10]
Time in ICU before intubation (days)	3 [0–6]
Smoker (*n*, %)	2 (7%)
Asthma (*n*, %)	2 (7%)
COPD (*n*, %)	4 (15%)
Berlin ARDS category:	
Moderate (100 < *P*/*F* ≤ 200) (*n*, %)	19 (70%)
Severe (*P*/*F* ≤ 100) (*n*, %)	8 (30%)
Body position at time of study	
Supine (*n*, %)	21 (78%)
Prone (*n*, %)	6 (22%)
ICU length of stay (days)	15 [10–24]*
Hospital length of stay (days)	20 [18–41]*
In hospital mortality (*n*, %)	11 (41%)

*BMI* body mass index, *APACHE III* acute physiology and chronic health evaluation, *COPD* chronic obstructive pulmonary disease, *ARDS* acute respiratory distress syndrome

^*^Five patients transferred to another hospital

**Table 2:** Pulmonary characteristics of the study cohort at baseline split by P_a_O_2_/F_i_O_2_ response

Variables	PEEP_low_ responders (*n* = 15)	PEEP_low_ non-responders (*n* = 12)	*p* value PEEP_low_	PEEP_high_ responders (*n* = 15)	PEEP_high_ non-responders (*n* = 12)	*p* value PEEP_high_
*Gas exchange*						
F_i_O_2_	0.62 ± 0.13	0.64 ± 0.13	0.69	0.62 ± 0.13	0.64 ± 0.13	0.69
P_a_O_2_ (mm Hg)	75 ± 18	81 ± 25	0.68	109 ± 21*	86 ± 25	< 0.001
P_a_O_2_/F_i_O_2_ ratio (mm Hg)	127 ± 41	138 ± 42	0.95	182 ± 41*	129 ± 58	0.02
P_a_CO_2_ (mm Hg)	65 ± 15	68 ± 16	0.83	66 ± 14	70 ± 10	0.98
ET-CO_2_ (mm Hg)	42 ± 11	45 ± 10	0.91	45 ± 12	49 ± 11	0.75
pH	7.27 ± 0.11	7.28 ± 0.09	0.98	7.26 ± 0.11	7.26 ± 0.09	0.99
BE (mmol/L)	1.2 ± 5.9	1.6 ± 4.2	0.89	1.0 ± 5.9	1.3 ± 4.4	0.91
*Pulmonary mechanics*						
*V*_t_ (mL/PBW kg)	6.5 ± 1.2	5.5 ± 2.7	0.39	6.5 ± 1.2	5.5 ± 2.7	0.58
RR (breaths/min)	19 ± 3.0	19 ± 2.2	0.99	19 ± 3.0	19 ± 2.2	0.99
*P*_plat_ (cm H_2_0)	27 ± 2.8	29 ± 2.7	0.78	34 ± 6.1*	38 ± 4.0*	0.01
PEEP (cm H_2_O)	11 ± 2.7	12 ± 3.2	0.58	18 ± 3.0*	18 ± 3.1*	0.99
*P*_dr_ (cm H_2_O)	15 ± 2.4	16 ± 1.8	0.78	9.5 ± 3.8*	17 ± 3.8	< 0.001
*C*_rs_ (mL/cm H_2_O)	27 ± 5	28 ± 9	0.70	34 ± 6*	25 ± 7	< 0.01

Values are mean ± standard deviation. The *p* values compare responders versus non-responders at PEEP_low_ and PEEP_high_

F_i_O_2_ = inspiratory fraction of oxygen; P_a_O_2_ = arterial partial pressure of oxygen; P_a_CO_2_ = arterial partial pressure of carbon dioxide; ET-CO_2_ = end-tidal carbon dioxide; BE = base excess; *V*_t_ = tidal volume; RR = respiratory rate; *P*_plat_ = plateau pressure; PEEP = positive end-expiratory pressure; *P*_dr_ = driving pressure; *C*_rs_ = compliance of the respiratory system

^*^Significant difference from PEEP_low_

**Fig. 1:**
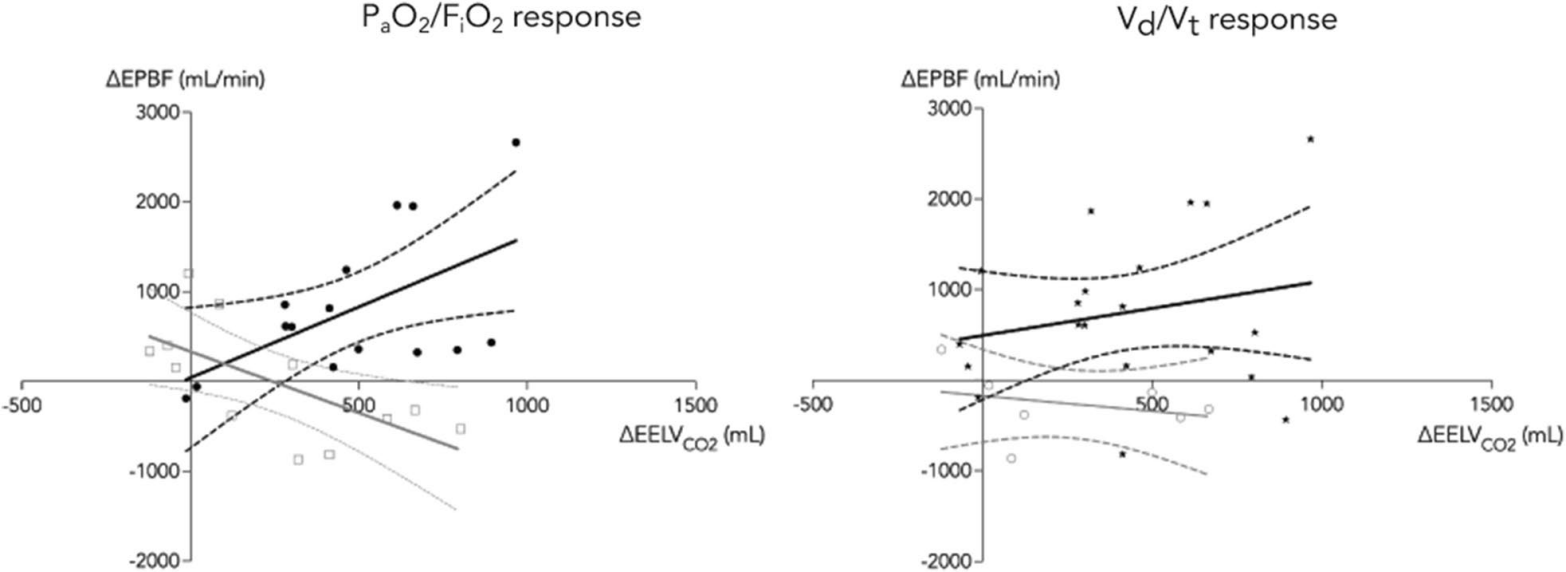
Capnodynamic monitoring of changes in end-expiratory lung volume (∆${\text{EELV}}_{{{\text{CO}}_{2} }}$) and effective pulmonary blood flow (∆EPBF) in patients with (solid dots, black lines) or without (open squares, grey lines) an increase in P_a_O_2_/F_i_O_2_ by > 20 mm Hg following increased PEEP level (left hand graph). Changes in end-expiratory lung volume (∆${\text{EELV}}_{{{\text{CO}}_{2} }}$) and effective pulmonary blood flow (∆EPBF) are also shown in patients with (stars, black lines) or without (open circles, grey lines) an improvement in *V*_d_/*V*_t_ following increased PEEP (right hand graph). Correlations are shown as Pearson’s regression (solid line) with the 95% confidence intervals (dashed lines)

The ${\text{EELV}}_{{{\text{CO}}_{2} }}$ was less in responders (1286 ± 347 ml) compared to non-responders (1746 ± 599 ml) at PEEP_low_ (mean difference 486 [88–831] mL, *p* = 0.01) but not at PEEP_high_ (1804 ± 462 mL in responders, 2052 ± 652 in non-responders, mean difference 241 [194–682], *p* = 0.61). These findings remained when ${\text{EELV}}_{{{\text{CO}}_{2} }}$ was indexed to body surface area (data not shown). The ${\text{EELV}}_{{{\text{CO}}_{2} }}$ indexed by PBW was 20 ± 5.7 mL/kg and 26 ± 6.5 mL/kg (mean difference 5.8 [1.0–8.5] mL/kg, *p* < 0.01) at PEEP_low_ and 28 ± 6.8 mL/kg and 31 ± 7.7 mL/kg (mean difference 2.7 [− 4.6 to 9.9] ml/kg, *p* = 0.73) at PEEP_high_ in responders and non-responders, respectively. The EBPF was not statistically different between non-responders and responders at PEEP_low_ (4.23 ± 1.67 L/min vs. 4.36 ± 1.59 L/min, *p* = 0.88) nor at PEEP_high_ (4.42 ± 1.61 L/min vs. 4.78 ± 1.61 L/min, *p* = 0.94). The concomitant changes in ${\text{EELV}}_{{{\text{CO}}_{2} }}$ and EPBF to increased PEEP in P_a_O_2_/F_i_O_2_ responders demonstrated a positive correlation (*r* = 0.56 [0.18–0.83], *p* = 0.03), i.e. recruitment of lung volume was associated with increased pulmonary perfusion (Fig. 1, left graph). In contrast, non-responders demonstrated a negative correlation (*r* = − 0.65 [− 0.12 to − 0.89], *p* = 0.02), i.e. an increased lung volume was associated with decreased pulmonary perfusion (Fig. 1, left graph). In P_a_O_2_/F_i_O_2_ responders, *V*_d_*V*_t_ decreased from PEEP_low_ (0.43 ± 0.12) to PEEP_high_ (0.36 ± 0.10) (mean difference − 0.06 [− 0.02 to − 0.09], *p* = 0.001), while no statistically significant difference was observed in non-responders (PEEP_low_ 0.40 ± 0.08 vs. PEEP_high_ 0.43 ± 0.10, *p* = 0.38). The *V*_d_/*V*_t_ responses were further explored by correlating changes in ${\text{EELV}}_{{{\text{CO}}_{2} }}$ and EBPF. For the 20 patients with reduced *V*_d_/*V*_t_, the increase in ${\text{EELV}}_{{{\text{CO}}_{2} }}$ was significantly correlated with increased or maintained EPBF (*r* = 0.46 [0.04–0.75], *p* = 0.04), while the correlation in seven patients with increased *V*_d_/*V*_t_ failed to attain statistical significance (*r* = − 0.29, *p* = 0.53) (Fig. 1, right graph).

**Fig. 2:**
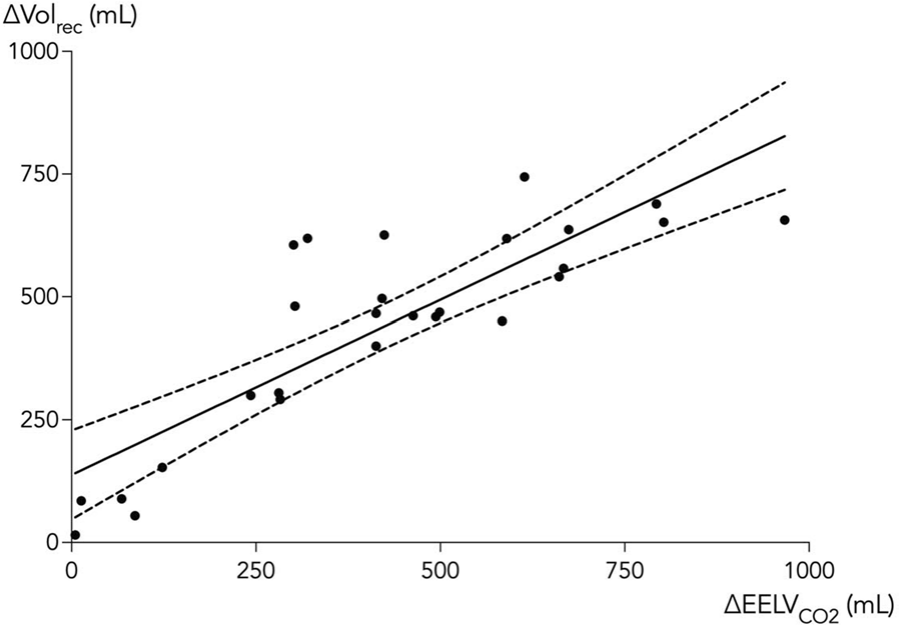
Changes in end-expiratory lung volume by capnodynamic monitoring (∆${\text{EELV}}_{{{\text{CO}}_{2} }}$) and the recruited lung volume (∆Vol_rec_) assessed based on the exhaled tidal volume at the rapid reduction from PEEP_high_ to PEEP_low_ as previously described [19]. The correlation is shown as Pearson’s regression (solid line) with the 95% confidence intervals (dashed lines)

**Fig. 3:**
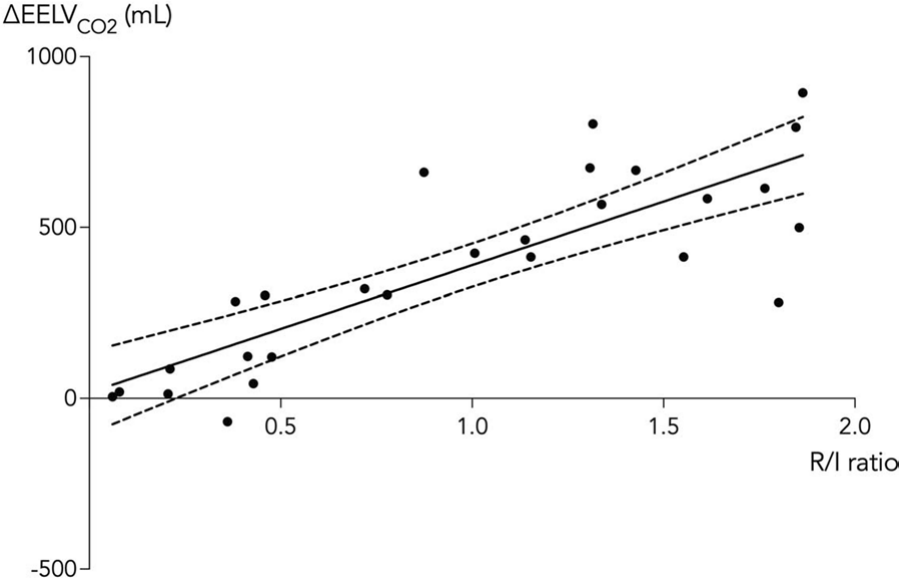
Changes in end-expiratory lung volume by capnodynamic monitoring (∆${\text{EELV}}_{{{\text{CO}}_{2} }}$) and the recruitment-to-inflation ratio assessed at the rapid reduction from PEEP_high_ to PEEP_low_ as previously described [19]. The correlation is shown as Pearson’s regression (solid line) with the 95% confidence intervals (dashed lines)

The change in ${\text{EELV}}_{{{\text{CO}}_{2} }}$ correlated with the ∆Vol_rec_ (*r* = 0.85 [0.69–0.93, *p* < 0.0001] (Fig. 2) and a positive correlation was demonstrated between the *R*/*I* ratio and the change in ${\text{EELV}}_{{{\text{CO}}_{2} }}$ from PEEP_low_ to PEEP_high_ (*r* = 0.87 [0.74–0.94], *p* ≤ 0.0001) (Fig. 3). The median *R*/*I* ratio for all 27 patients was 1.0 and the PEEP induced change in ${\text{EELV}}_{{{\text{CO}}_{2} }}$ below and above the median *R*/*I* ratio was 170 ± 198 mL and 578 ± 176 mL, respectively (mean difference 408 [236–546] mL, *p* < 0.0001).

## Discussion

In this pragmatic, observational open study of capnodynamic monitoring in mechanically ventilated patients with moderate to severe C-ARDS, an improved P_a_O_2_/F_i_O_2_ ratio in response to increased PEEP, was associated with increased end-expiratory lung volume and pulmonary perfusion. The change in end-expiratory lung volume was positively correlated with the lung volume recruited and the recruitment-to-inflation ratio. In patients without an improvement in P_a_O_2_/F_i_O_2_ ratio, PEEP increased end-expiratory lung volume with a decrease lung perfusion consistent with increased dead space. This study demonstrates the feasibility of capnodynamic monitoring to assess physiological responses to PEEP at the bedside to facilitate an individualised setting of PEEP.

Patients were studied about a week after their COVID-19 diagnosis with the majority developing moderate ARDS. A majority of patients in this study improved the P_a_O_2_/F_i_O_2_ > 20 mm Hg in response to increased PEEP. Compared to recent observational reports of PEEP interventions in C-ARDS, the *C*_rs_ was similarly low [7, 22] or lower [6, 23, 24] with *P*_dr_ correspondingly higher. Patients in this study were class 2 obese with half having a body mass index above 35. This suggests that the prevalence and degree of obesity leading to an increased load on the chest wall should be considered together with the reduced lung compliance associated with C-ARDS. A lung protective ventilation strategy limiting airway pressures was employed including permissive hypercapnia. The associated moderate respiratory acidosis might have aggravated pulmonary vasoconstriction. The changes in ${\text{EELV}}_{{{\text{CO}}_{2} }}$ and EPBF in response to increased PEEP should be interpreted with those characteristics of the study cohort in mind.

The ${\text{EELV}}_{{{\text{CO}}_{2} }}$ at PEEP_low_ (mean PEEP 8 cm H_2_O) was overall similar to the range of end-expiratory lung volumes, 1000–1400 mL, at PEEP 5–8 cm H_2_O reported in C-ARDS [6, 7, 22, 24] and non-COVID ARDS [25] using chest computed tomography. The EPBF, that does not include shunt flow, was numerically consistent with a normal cardiac output reflecting the inclusion criterion of haemodynamic stability prior to study procedures. The increased P_a_O_2_/F_i_O_2_ in response to PEEP_high_ was associated with increases in both ${\text{EELV}}_{{{\text{CO}}_{2} }}$ and EPBF and this positive correlation supports an improved ventilation/perfusion matching. The greater ${\text{EELV}}_{{{\text{CO}}_{2} }}$ is consistent with recruitment of previously non-aerated pulmonary tissue that is in line with the concomitant improvement in *C*_rs_ and decrease in *P*_dr_. Importantly, a reduced shunt fraction would result in an increased EPBF and this plausibly explains the observed response in gas exchange to PEEP_high_. A PEEP_high_-induced decrease in cardiac output from the typical hyperdynamic haemodynamic state of C-ARDS [26, 27] would reduce the shunt fraction as would recruitment of previously perfused but not ventilated lung areas. The increase in EPBF could furthermore indicate a maintained or potentially increased cardiac output as PEEP_high_ reduced pulmonary vascular resistance along with decreased atelectases. A previous study of C-ARDS patients who underwent pulmonary artery catheterisation reported an inverse relation between P_a_O_2_/F_i_O_2_ and shunt at both low (5 cm H_2_O) and high (15 cm H_2_O) PEEP levels without a significant reduction in cardiac output [28]. The improved ventilation/perfusion matching is also supported by the reduced dead space observed in responders. In contrast, patients without a significant improvement of P_a_O_2_/F_i_O_2_ in response to PEEP_high_ demonstrated an increased ${\text{EELV}}_{{{\text{CO}}_{2} }}$ but decreased EPBF. This is consistent with overstretching the lungs, increased pulmonary vascular resistance and right ventricular strain that would reduce pulmonary perfusion. While these changes point to increased dead space, the numerical increase in *V*_d_/*V*_t_ and the negative correlation between ${\text{EELV}}_{{{\text{CO}}_{2} }}$ and EPBF failed, however, to attain statistical significance.

The significant correlation between the change in ${\text{EELV}}_{{{\text{CO}}_{2} }}$ and the independently measured ∆Vol_rec_ in response to PEEP lends support to the validity of capnodynamic monitoring of lung volumes in C-ARDS. The correlation coefficient was similar to that reported between absolute ${\text{EELV}}_{{{\text{CO}}_{2} }}$ and functional residual capacity in a porcine experimental model [29] and superior to that previously reported in anaesthetised patients [14]. Since tidal volumes in this study were kept unchanged from PEEP_low_ to PEEP_high_, the increased ${\text{EELV}}_{{{\text{CO}}_{2} }}$ represents a true recruitment effect. In 6 patients, the ${\text{EELV}}_{{{\text{CO}}_{2} }}$ failed to increase during PEEP_high_ using a threshold of at least + 10% to consider random measurements error. This represents a lack of alveolar recruitment where additional PEEP contributes to increased lung stress without any benefit in gas exchange. The capacity of capnodynamic monitoring at the bedside to facilitate an individualised setting of PEEP warrants further clinical investigation to evaluate if it can contribute to minimising ventilator induced lung injury in C-ARDS and non-COVID ARDS [30].

Haemodynamic changes may affect ${\text{EELV}}_{{{\text{CO}}_{2} }}$ since CO_2_ kinetics are dependent on pulmonary blood flow. Experimental observations, however, demonstrate ${\text{EELV}}_{{{\text{CO}}_{2} }}$ and EPBF as independent factors in the capnodynamic equation [29] in a wide range of cardiac output states. Within this study, three sets of observations were made for patients who progressed to veno-venous extracorporeal membrane oxygenation support (Additional File 1: Fig. S1). The ${\text{EELV}}_{{{\text{CO}}_{2} }}$ remained stable during variable pump flow and native pulmonary perfusion states that corroborates the potential to separately monitor ${\text{EELV}}_{{{\text{CO}}_{2} }}$ and EPBF by the capnodynamic algorithm.

The change in ${\text{EELV}}_{{{\text{CO}}_{2} }}$ was also significantly correlated to alveolar recruitment as indicated by the *R*/*I* ratio. The median *R*/*I* ratio of 1 was higher compared to other studies reporting a median around 0.7 [10, 31] and higher than the threshold of 0.5 previously used to differentiate poorly from highly recruitable patients in C-ARDS [23, 32] and non-COVID ARDS [19]. Recruitability in acute respiratory failure may be highly variable between patients and over time. In this study, a similar proportion (17/27; 63%) of patients would have been considered highly recruitable by an *R*/*I* ratio > 0.5 compared to what has been reported in patients intubated early after ICU admission [23] but higher than that in patients intubated late [32]. Most patients in this study demonstrated low compliance and high recruitability consistent with the high elastance (“H”) phenotype based on recruitability idiosyncratic to C-ARDS [33]. More recent studies have questioned this distinction and instead reported similar patterns in C-ARDS and non-COVID ARDS [34]. Irrespectively, capnodynamic monitoring allowed changes in functional lung volume to be continuously monitored during manoeuvres aimed at alveolar recruitment in C-ARDS.

This study has some important limitations. External validity might be limited by the relatively small sample size, non-consecutive enrolment dependent on availability of the clinical research team and a high proportion of responders to recruitment by increased PEEP. No standard comparators were included for ${\text{EELV}}_{{{\text{CO}}_{2} }}$ or EPBF since this pragmatic study was primarily designed to evaluate the feasibility of capnodynamic monitoring and validation studies have already been published [14, 29]. Levels of PEEP above the PEEP_high_ might be considered for alveolar recruitment but were not investigated for effects on ${\text{EELV}}_{{{\text{CO}}_{2} }}$ and EPBF. The R/I ratio was not measured from the 15–5 cm H_2_O pressure drop as originally described [19] with less of a pressure difference achieved between PEEP_high_ and PEEP_low_. A formal assessment of airway opening pressure was not performed. Visual inspection, however, confirmed progressive, steep increases in both volume and pressure curves from the start of a breath.

## Conclusion

This pragmatic, observational open study using capnodynamic monitoring in patients with COVID-19 ARDS demonstrated associations between an improved P_a_O_2_/F_i_O_2_ ratio in response to increased PEEP and increased end-expiratory lung volume and pulmonary perfusion. The change in end-expiratory lung volume was positively correlated with independent measures of recruited lung volume and the recruitment-to-inflation ratio. Capnodynamic monitoring is feasible to assess physiological responses to PEEP at the bedside and could facilitate an individualised level of PEEP during mechanical ventilatory support.

## Supplementary Information

**Additional file 1** STROBE checklist and ECMO cases.

## Data Availability

Anonymised data are available upon reasonable request and if approved by the South Western Sydney Local Health District Human Research Ethics Committee. No custom code was used and software for statistical analysis is stated in Methods and available in the public domain.

## Abbreviations

∆Vol_rec_: Recruited lung volume
C-ARDS: COVID-19 acute respiratory distress syndrome
*C*_rs_: Static compliance of the respiratory system
C_v_CO_2_: Mixed venous carbon dioxide content
ECMO: Extracorporeal membrane oxygenation
${\text{EELV}}_{{{\text{CO}}_{2} }}$: End-expiratory lung volume by capnometry
EPBF: Effective pulmonary blood flow
ET-CO_2_: End-tidal carbon dioxide
F_A_CO_2_: Alveolar fraction of carbon dioxide
F_i_O_2_: Inspiratory fraction of oxygen
IQR: Interquartile range
P_a_CO_2_: Arterial partial pressure of carbon dioxide
P_a_O_2_: Arterial partial pressure of oxygen
P_a_O_2_/F_i_O_2_: Arterial partial oxygen pressure to inspired oxygen fraction ratio
PBW: Predicted body weight
*P*_dr_: Driving pressure
PEEP: Positive end-expiratory pressure
*P*_plat_: Plateau pressure
*R*/*I* ratio: Recruitment-to-inflation ratio
RR: Respiratory rate
SD: Standard deviation
S_p_O_2_: Peripheral oxygen saturation
*V*_d_/*V*_t_: Physiological dead space
*V*_t_: Tidal volume
