# Supplementary material for: Capnodynamic monitoring of lung volume and blood flow in response to increased positive end-expiratory pressure in moderate to severe COVID-19 pneumonia: an observational study
Source: Crit Care. 2022 Jul 31;26:232. doi: 10.1186/s13054-022-04110-0 (PMC9340710; doi:10.1186/s13054-022-04110-0)

**ADDITIONAL FILE**

**Table 1.** STROBE checklist.

|  | Item No | Recommendation | Page No |
| --- | --- | --- | --- |
| **Title and abstract** | 1 | (*a*) Indicate the study’s design with a commonly used term in the title or the abstract | Page 1, lines 1-3 |
|  |  | (*b*) Provide in the abstract an informative and balanced summary of what was done and what was found | Page 2, line 33-page 3, line 51 |
| Introduction | | | |
| Background/  rationale | 2 | Explain the scientific background and rationale for the investigation being reported | Page 4, lines 62-83 |
| Objectives | 3 | State specific objectives, including any prespecified hypotheses | Page 4, line 83-page 5, line 88 |
| Methods | | | |
| Study design | 4 | Present key elements of study design early in the paper | Page 5, line 91 |
| Setting | 5 | Describe the setting, locations, and relevant dates, including periods of recruitment, exposure, follow-up, and data collection | Page 5, lines 93-94 |
| Participants | 6 | (*a*) Give the eligibility criteria, and the sources and methods of selection of participants. Describe methods of follow-up | Page 5, lines 98-99, page 5 line 106-page 6 line 115 |
|  |  | (*b*) For matched studies, give matching criteria and number of exposed and unexposed | NA |
| Variables | 7 | Clearly define all outcomes, exposures, predictors, potential confounders, and effect modifiers. Give diagnostic criteria, if applicable | Page 5, lines 99-106, page 6, lines 117-123, page 6 line 132-page 7 line 137, page 9 lines 180-184 |
| Data sources /measurement | 8* | For each variable of interest, give sources of data and details of methods of assessment (measurement). Describe comparability of assessment methods if there is more than one group | Page 7 line 142-page 8 line 178 |
| Bias | 9 | Describe any efforts to address potential sources of bias | NA |
| Study size | 10 | Explain how the study size was arrived at | Page 9, lines 186-188 |
| Quantitative variables | 11 | Explain how quantitative variables were handled in the analyses. If applicable, describe which groupings were chosen and why | Page 9, lines 181-182 |
| Statistical methods | 12 | (*a*) Describe all statistical methods, including those used to control for confounding | Page 9, lines 186-197 |
|  |  | (*b*) Describe any methods used to examine subgroups and interactions | NA |
|  |  | (*c*) Explain how missing data were addressed | NA |
|  |  | (*d*) If applicable, explain how loss to follow-up was addressed | NA |
|  |  | (*e*) Describe any sensitivity analyses | NA |
| Results | | |  |
| Participants | 13* | (a) Report numbers of individuals at each stage of study—eg numbers potentially eligible, examined for eligibility, confirmed eligible, included in the study, completing follow-up, and analysed | Page 9 line 200-page 10 line 205 |
|  |  | (b) Give reasons for non-participation at each stage |  |
|  |  | (c) Consider use of a flow diagram |  |
| Descriptive data | 14* | (a) Give characteristics of study participants (eg demographic, clinical, social) and information on exposures and potential confounders | Table 1 |
|  |  | (b) Indicate number of participants with missing data for each variable of interest |  |
|  |  | (c) Summarise follow-up time (eg, average and total amount) |  |
| Outcome data | 15* | Report numbers of outcome events or summary measures over time | Tables 2 |

| Main results | 16 | (*a*) Give unadjusted estimates and, if applicable, confounder-adjusted estimates and their precision (eg, 95% confidence interval). Make clear which confounders were adjusted for and why they were included | Page 10 line 205-page 11 line 243 |
| --- | --- | --- | --- |
|  |  | (*b*) Report category boundaries when continuous variables were categorized |  |
|  |  | (*c*) If relevant, consider translating estimates of relative risk into absolute risk for a meaningful time period |  |
| Other analyses | 17 | Report other analyses done—eg analyses of subgroups and interactions, and sensitivity analyses | NA |
| Discussion | | | |
| Key results | 18 | Summarise key results with reference to study objectives | Page 11 line 246-page 12 line 254 |
| Limitations | 19 | Discuss limitations of the study, taking into account sources of potential bias or imprecision. Discuss both direction and magnitude of any potential bias | Page 12, lines 256-267, page 15, lines 331-342 |
| Interpretation | 20 | Give a cautious overall interpretation of results considering objectives, limitations, multiplicity of analyses, results from similar studies, and other relevant evidence | Page 15 line 345-page 16 line 352 |
| Generalisability | 21 | Discuss the generalisability (external validity) of the study results | Page 12 line 269-page 15 line 329 |
| Other information | | | |
| Funding | 22 | Give the source of funding and the role of the funders for the present study and, if applicable, for the original study on which the present article is based | Page 18, lines 379-382 |

**Figure 1.** In three patients supported by veno-venous extracorporeal membrane oxygenation (ECMO) using femoral drainage and jugular return cannulae, capnodynamic monitoring was performed during weaning studies. The flow in the ECMO circuit was increased or decreased by 20-40% while the end-expiratory lung volume (EELV_CO2_) remained stable.


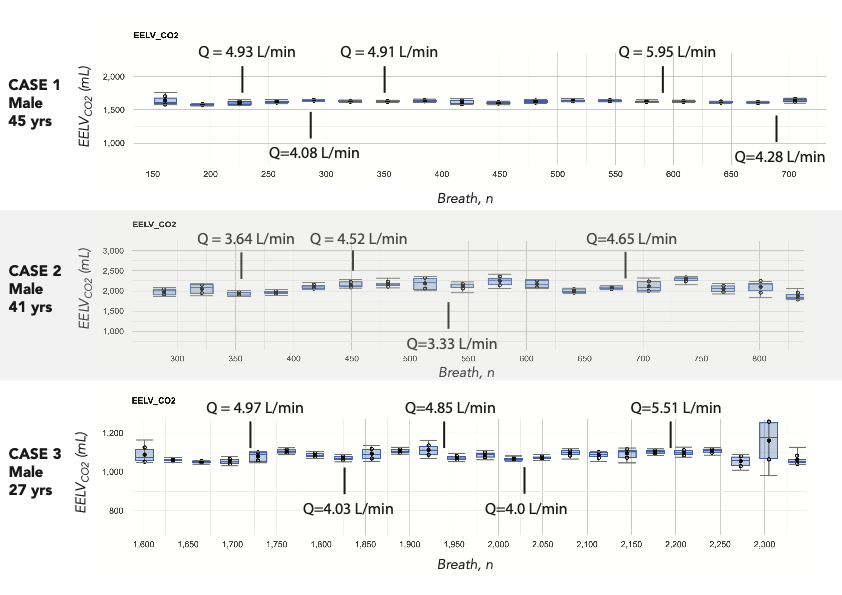

Supplement: Supplementary file 1 — Additional file 1 STROBE checklist and ECMO cases. [file 13054_2022_4110_MOESM1_ESM.docx]
